# Management of metastatic endometrial cancer: physicians’ choices beyond the first line after approval of checkpoint inhibitors

**DOI:** 10.3389/fonc.2023.1247291

**Published:** 2023-09-14

**Authors:** Francesca Arezzo, Gaia Giannone, Daniele Castaldo, Giulia Scotto, Valentina Tuninetti, Margherita Turinetto, Michele Bartoletti, Serafina Mammoliti, Grazia Artioli, Giorgia Mangili, Vanda Salutari, Domenica Lorusso, Gennaro Cormio, Vera Loizzi, Claudio Zamagni, Antonella Savarese, Massimo Di Maio, Graziana Ronzino, Carmela Pisano, Sandro Pignata, Giorgio Valabrega

**Affiliations:** ^1^ Gynecologic Oncology Unit, IRCCS Istituto Tumori “Giovanni Paolo II”, Bari, Italy; ^2^ Department of Precision and Regenerative Medicine – Ionian Area, University of Bari “Aldo Moro”, Bari, Italy; ^3^ Department of Surgery and Cancer, Imperial College London, London, United Kingdom; ^4^ Segreteria Multicenter Italian Trials in Ovarian Cancer and Gynecologic Malignancies (MITO) Group, Naples, Italy; ^5^ Department of Oncology, University of Turin, Turin, Italy; ^6^ Department of Oncology, Mauriziano Hospital, University of Turin, Turin, Italy; ^7^ Unit of Medical Oncology and Cancer Prevention, Department of Medical Oncology, Centro di Riferimento Oncologico di Aviano (CRO), IRCCS, Aviano, Italy; ^8^ Ospedale Policlinico San Martino - Department of Medical Oncology, IRCCS, Genoa, Italy; ^9^ Oncologia Medica, Unità locale socio sanitaria n2 (ULSS2) Marca Trevigiana, Treviso, Italy; ^10^ Obstet-Gynecol Department, San Raffaele Scientific Institute, IRCCS, Milan, Italy; ^11^ Department of Women and Child Health, Division of Gynecologic Oncology, Fondazione Policlinico Universitario A. Gemelli, IRCCS, Rome, Italy; ^12^ Department of Life Science and Public Health, Catholic University of Sacred Heart Largo Agostino Gemelli, and Fondazione Policlinico Universitario A. Gemelli, IRCCS, Rome, Italy; ^13^ Interdisciplinar Department of Medicine, University of Bari “Aldo Moro”, Bari, Italy; ^14^ Azienda Ospedaliero-universitaria di Bologna, IRCCS, Bologna, Italy; ^15^ Department of Oncology, IRCCS Regina Elena National Cancer Institute, Rome, Italy; ^16^ Department of Oncology, Ospedale “Vito Fazzi”, Lecce, Italy; ^17^ Department of Urology and Gynecology, Istituto Nazionale Tumori IRCCS Fondazione G. Pascale Napoli, Naples, Italy

**Keywords:** endometrial cancer, molecular classification, immune checkpoint inhibitors, dostarlimab, pembrolizumab plus lenvatinib

## Abstract

**Introduction:**

Endometrial cancer (EC) represents 3.4% of all newly diagnosed cancer cases and is responsible for 2.1% of all cancer-related deaths. Approximately 10%–15% of women with EC are diagnosed with advanced-stage disease, resulting in a reported 5-year survival rate of only 17% for those with distant metastases. A better understanding of its molecular features has ushered in a new era of immunotherapy for the treatment of EC, allowing for alternative therapeutic approaches, even in cases of advanced disease.

**Methods:**

We administered a multi-choice online survey for Multicenter Italian Trials in Ovarian cancer and gynecologic malignancies (MITO) members. The questionnaire was available for 2 months, starting in October 2022. Our objective was to evaluate the current attitude of incorporating molecular characterization of EC into routine clinical practice, appraise the implementation of newly available therapies, and compare the outcomes with the previous survey conducted in April–May 2021 to ascertain the actual changes that have transpired during this recent time period.

**Results:**

The availability of molecular classification in Italian centers has changed in 1 year. Seventy-five percent of centers performed the molecular classification compared with 55.6% of the previous survey. Although this percentage has increased, only 18% performed all the tests. Significant changes have occurred in the administration of new treatments in EC patients in MITO centers. In 2022, 82.1% of the centers administrated dostarlimab in recurrent or advanced MMR-deficient (dMMR) EC experiencing disease progression after platinum-based chemotherapy regimens, compared to only 24.4% in 2021. In 2022, 85.7% of the centers already administrated the pembrolizumab plus lenvatinib combination as a second-line therapy for MMR-proficient (pMMR) patients with advanced or recurrent EC who had progressed from first-line platinum-based therapy.

**Conclusion:**

Both the therapeutic and diagnostic scenarios have changed over the last couple of years in MITO centers, with an increased prescription of immune checkpoint inhibitors and use of the molecular classification.

## 1 Introduction

It has been estimated that 417,367 new cases of endometrial cancer (EC) have been diagnosed worldwide, causing the deaths of 97,370 women in 2020 (1). According to the National Cancer Institute, it represents 3.4% of all new cancer cases and causes 2.1% of all cancer deaths (2). The 5-year survival rate for EC is 81.3% because it is usually diagnosed at an early stage due to symptoms such as postmenopausal bleeding, but women with advanced or recurring EC have a dismal prognosis (3).

Indeed, 10%–15% of women diagnosed with EC have advanced-stage disease at presentation, with reported 5-year survival rates of only 17% for those with distant metastases (2). Until a few years ago, the therapeutic armamentarium was quite limited and for patients who had progressed after a first-line platinum-based chemotherapy, effective strategies were lacking (3).

Endocrine therapy could be considered, particularly for patients with lower-grade disease and a prolonged time to recurrence (4), while in second and further lines, few therapeutic options are available with low chances of response (5).

Moreover, the prognostic and predictive role of molecular features was not well known. During the last decade, both the diagnostic and therapeutic approach in these patients changed dramatically. First, the integration of molecular characterization of EC into the European (ESGO-ESTRO-ESP) guidelines changed the management of patients with early-stage EC (5). This revolution started with the Cancer Genome Atlas (TCGA) Research Network identifying four molecular prognostic groups that could be replicated using a mixture of immunohistochemistry (IHC) and hotspot sequencing. This paved the way for their use in the clinic, giving valuable information on how to tailor adjuvant treatment, and it has been recommended by ESGO/ESTRO/ESP guidelines in 2020 (5).

These four groups are DNA Polymerase Epsilon (POLE)-mutated, mismatch repair (MMR)-deficient (dMMR), p53-abnormal, and “no specific molecular profile” (NSMP) (6). The first two are characterized by an impairment in DNA repair, with high mutational rate and neoantigen load. These two subgroups have also abundance of tumor-infiltrating lymphocytes and increased expression of programmed cell death protein 1 (PD-1) and PD-L1, making them inflamed and potentially more susceptible to immune checkpoint inhibition (7).

From a therapeutic point of view, the introduction of immune checkpoint inhibitors have indeed revolutionized the treatment of advanced EC (8).

First, dostarlimab, a humanized monoclonal antibody targeting PD1, has been tested in a phase I study, with an objective response rate of 42.3% in dMMR recurrent or advanced EC patients in second or further lines (9).

As a result, in April 2021, dostarlimab was granted accelerated approval by the U.S. Food and Drug Administration (FDA) for the treatment of patients with recurrent or advanced dMMR EC experiencing disease progression after treatment with platinum-containing chemotherapy regimens (10).

This was followed by results from the KEYNOTE-775 study, which compared the efficacy and safety of lenvatinib plus pembrolizumab versus the administration of doxorubicin or paclitaxel chemotherapy in women with advanced EC who had disease progression after at least one platinum-based therapy (11). The trial demonstrated that both progression-free survival (PFS) and overall survival (OS) were significantly longer with lenvatinib plus pembrolizumab compared to chemotherapy, in both the MMR-proficient (pMMR) population and all patients, with a median OS that is 5.4 months longer in pMMR (hazard ratio for death, 0.68; 95% CI, 0.56 to 0.84; *p* < 0.001) and 6.9 months longer in overall population (hazard ratio, 0.62; 95% CI, 0.51 to 0.75; *p* < 0.001) (12).

A Multicenter Italian Trials in Ovarian cancer and gynecologic malignancies (MITO) survey, performed during April–May 2021, showed that more than half of the highly specialized centers in Italy performed the molecular classification, but only 13.3% of these ran all the tests needed for it. At the same time, 80% of respondents declared regular assessment of MSI status with IHC. In 2021, the most frequent choice in second line has been chemotherapy (53.3%) and dostarlimab was administrated in only 24.4% of centers. Furthermore, for MSS patients, 77.8% of clinicians stated that they would choose lenvatinib plus pembrolizumab in second line once approved (13).

We aim at evaluating the changes both in the therapeutic and the diagnostic algorithm during the last year, comparing the current scenario with the results of the previous survey (13).

The results of recent studies investigating the administration of checkpoint inhibition combined with chemotherapy have shown highly promising outcomes. Specifically, the ENGOT-EN6-NSGO/GOG-3031/RUBY trial has provided support for the utilization of dostarlimab in combination with chemotherapy. The study demonstrated statistically significant and clinically relevant benefits PFS for both the dMMR/MSI-H population and the overall population when compared to treatment with carboplatin-paclitaxel alone. Additionally, in the pMMR/MSS population, a clinically relevant improvement in PFS was observed, along with an early trend suggesting improved OS (14). Furthermore, NRG-GY018 showed that the addition of pembrolizumab to standard chemotherapy resulted in significantly longer PFS than with chemotherapy alone either in the dMMR/MSI-H cohort or in the pMMR/MSS cohort (15). Therefore, the combination of checkpoint inhibition and carboplatin-paclitaxel represents a new standard of care for patients with newly diagnosed primary advanced or recurrent EC (16).

## 2 Methods

To assess how much these changes have impacted the management of patients with EC, we led a survey among MITO centers. The survey was available online on the MITO website only for MITO members from 20 October 2022 to 20 December 2022. The questionnaire was realized by GV, GG, and FA; reviewed and discussed by the MITO scientific committee; and submitted to and approved by the MITO internal review board. The survey was composed of 25 multiple choice questions (see the list of questions in **Supplementary Table S1**). The first six questions were about geographical distributions and work experience of participants, four questions were about number of diagnosis and number of patients treated in each center, and three focused on the diagnostic algorithm and the performance of molecular characterization. The remaining questions were about the administration of immune checkpoint inhibitors and the referral to a genetic counselor. We analyzed one answer form per center. All replies were anonymized. Descriptive analyses are detailed in the Results session.

## 3 Results

An invitation to fill in the survey was sent to 166 MITO centers. Among this, 35 clinicians (4.9%) completed the survey. In seven cases, more than one respondent per center was recorded and we analyzed only one questionnaire per center. A total of 28 responses (16.9% of the MITO centers) were therefore analyzed (see details in **Supplementary Table S2**).

Features of the respondents are listed in **Table 1**. Most respondents were aged 40 or more (18/28, 64.3%), worked in a public hospital (13/28, 46.4%) or university hospital (9/28, 32.1%), and were located in the North of Italy (20/28, 71.4%) at the time of the survey. Most of the participants were medical oncologists (22/28, 78.6%) and treated mainly gynecological malignancies (23/28, 82.1%). They treated patients with gynecological cancers for a mean of 14.1 ± 8.5 years.

**Table 1:** Survey respondents’ characteristics.

Respondents’ characteristics			
	Mean (±SD)	Number	Percentage
Age			
<40 years old		10	35.7
>40 years old		18	64.3
Years in practice	14.1 ± 8.5 years		
Health organizations			
Public hospital		13	46.4
University hospital		9	32.1
Italian institutes for research and care		6	21.5
Location of the hospital			
North of Italy		20	71.4
Center of Italy		5	17.9
South of Italy		2	7.1
Islands		1	3.6
Medical training			
Medical oncology		22	78.6
Gynecology		6	21.4
Clinical focus			
Only gynecological cancers		3	10.7
Mainly gynecological cancers		23	82.1
Marginally gynecological cancers		2	7.2
Cumulative number of new EC diagnoses per month			
Less than 5		5	17.9
5–10		17	60.7
11–25		0	0
More than 25		6	21.4
Cumulative number of recurrent, locally advanced (unresectable), or metastatic EC patients treated per month			
Less than 5		6	21.4
5–10		12	42.9
11–25		10	35.7
Cumulative number of pretreated metastatic EC patients treated per month			
Less than 5		8	28.6
5–10		11	39.3
11–25		9	32.1

More than half of the centers (17/28, 60.7%) had 5 to 10 new EC diagnoses per month and 21.4% (6/28) recorded more than 25 new cases per month.

In second and further lines, 42.9% of centers (12/28) treated 5 to 10 patients and 35.7% (10/28) treated more than 10 patients per month.

Twenty participants (71.4%) did not have available clinical trials in second line for this subset of patients.

In almost all centers (26/28, 92.9%) was the status of estrogen and/or progesterone receptors assessed using IHC.

**Figure 1:**
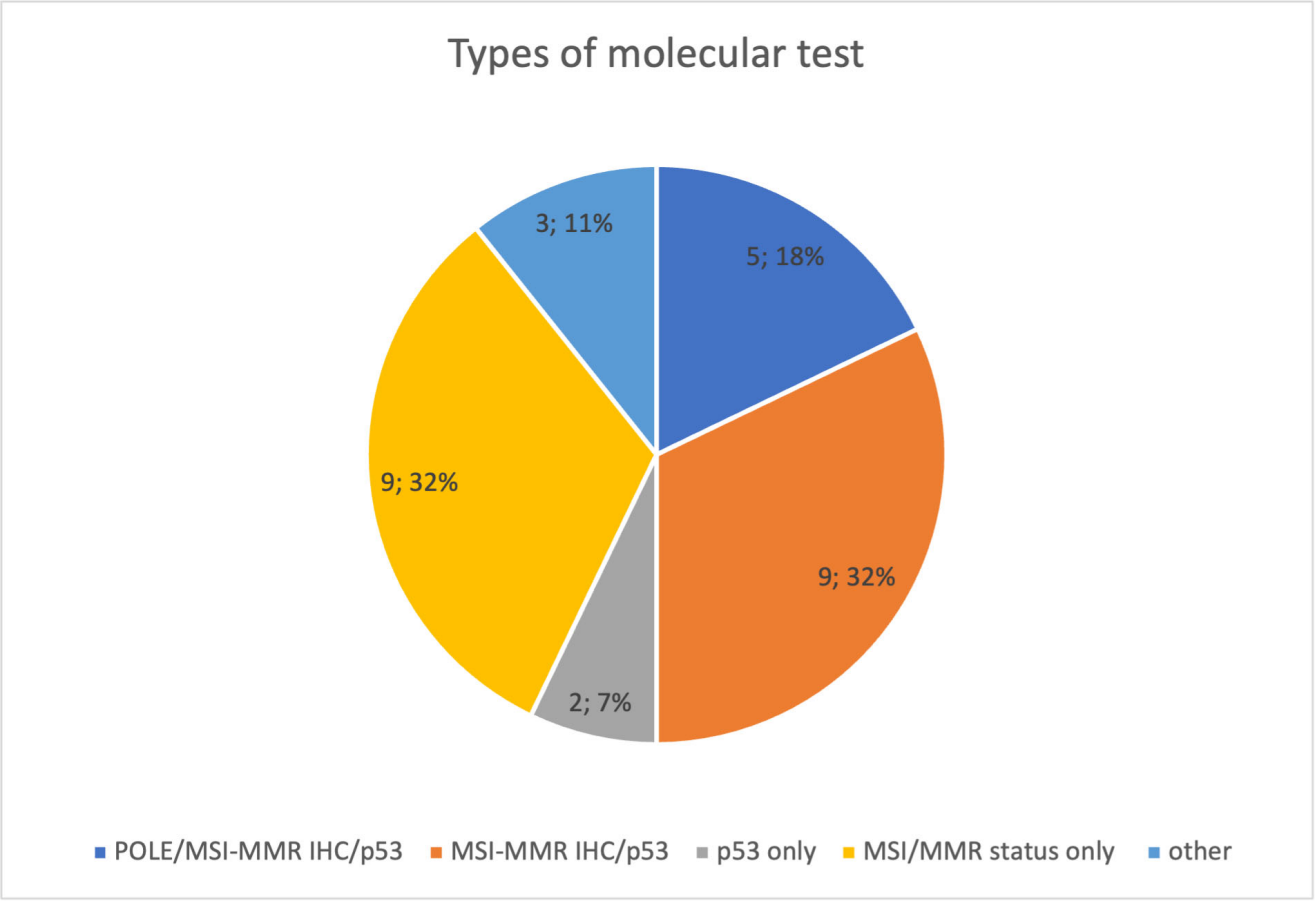
Types of molecular test performed in Multicenter Italian Trials in Ovarian cancer and gynecologic malignancies (MITO) centers.

Approximately 75% of respondents stated that their center performs the molecular characterization, but only 5 of 28 (18%) did all the tests [POLE hotspots sequencing, IHC for MMR proteins, or MSI status defined using polymerase chain reactions (PCRs) and p53 IHC]. Nine of 28 centers (32%) evaluated p53 and MMR proteins using IHC. p53 IHC was performed by only 2/28 (7%) and the evaluation of MSI/MMR status was performed by only 9/28 (32%). Three of 28 (11%) answered that they used other tests (unspecified) (**Figure 1**).

The status of MSI/MMR was evaluated performing IHC for all the four proteins (MLH1, MSH2, MSH6, and PMS2) in 21/28 (75%) centers and for MSH6 and PMS2 in only 1/28 (3.6%) centers. Four respondents (14.3%) used polymerase chain reaction (PCR) as a second step approach for indeterminate cases at IHC while it was performed upfront in 2/28 (7.1%) centers. Only 4/28 (14.3%) participants evaluated MLH1 methylation status.

**Figure 2:**
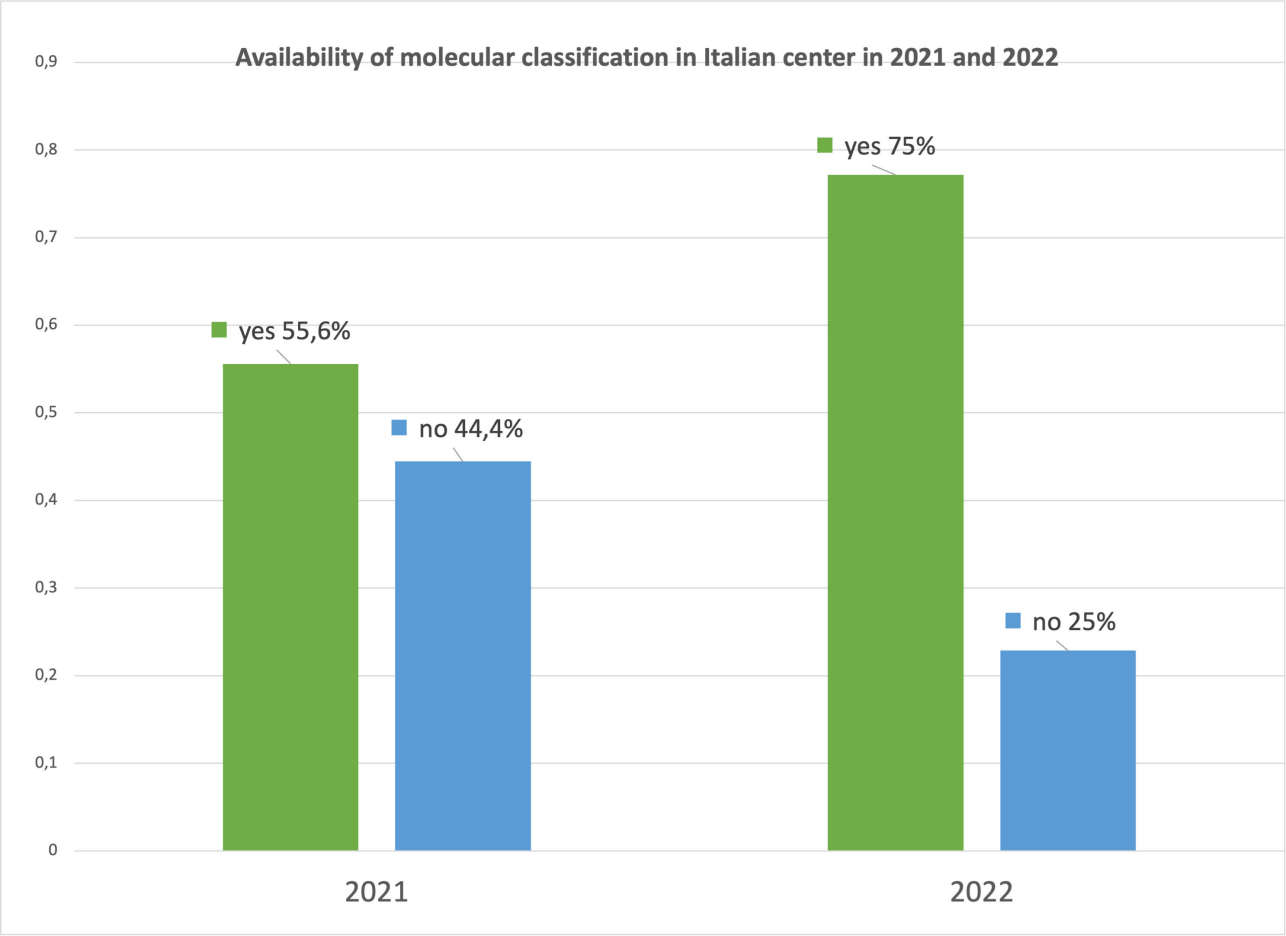
Availability of molecular classification in Multicenter Italian Trials in Ovarian cancer and gynecologic malignancies (MITO) centers in 2021 and 2022.

We compared the responses of the latest survey with the results recorded 1 year ago to evaluate if any change has occurred. Seventy-five percent of centers performed the molecular classification in 2022 compared with 55.6% of the previous survey with an increase of approximately 20% (**Figure 2**).

**Figure 3:**
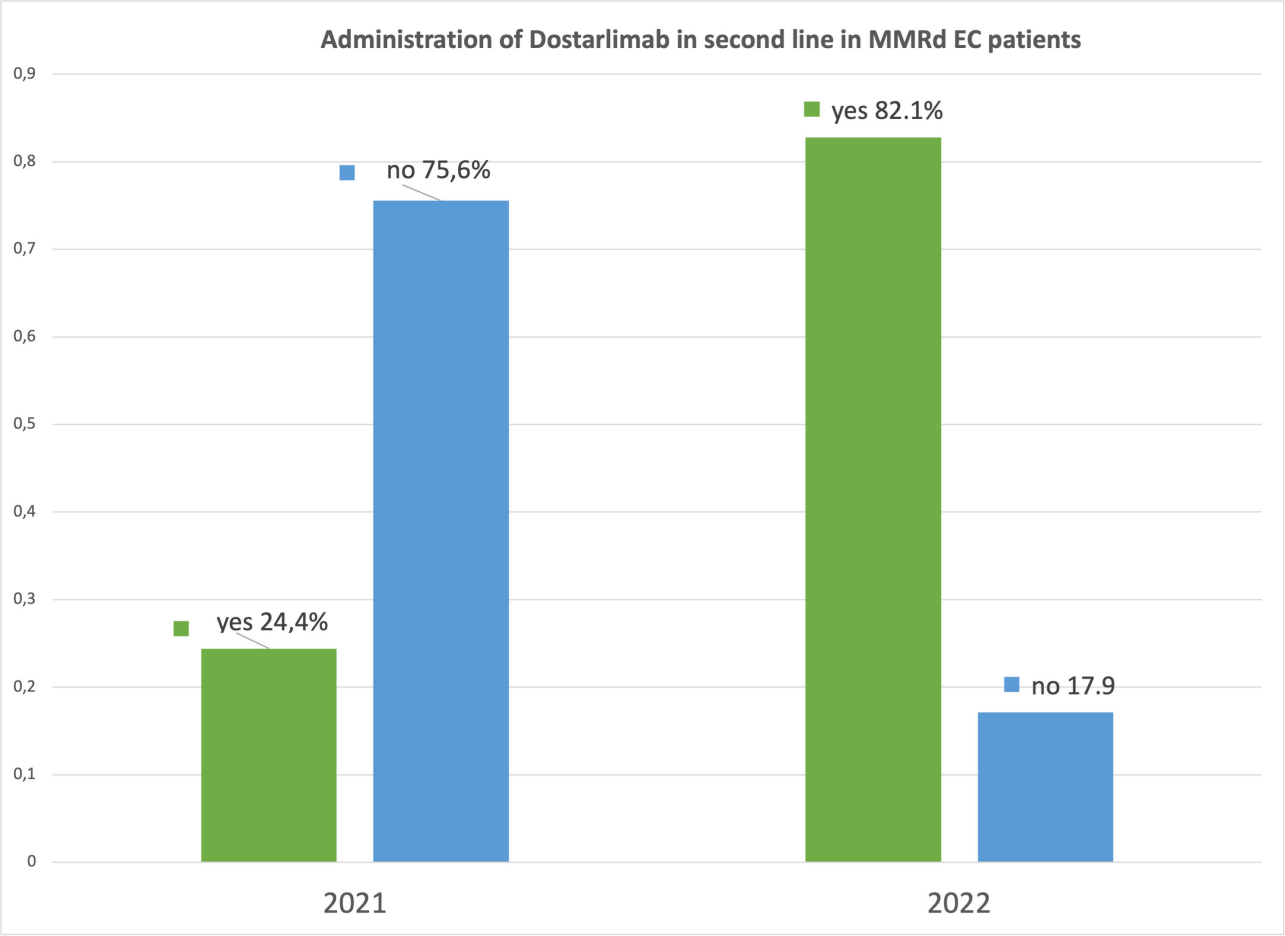
Administration of dostarlimab in Multicenter Italian Trials in Ovarian cancer and gynecologic malignancies (MITO) centers in 2021 and 2022.

The change observed in 1 year regarding the administration of dostarlimab in dMMR EC in the different Italian MITO centers is significant. In 2021, only 24.4% clinicians had one to five patients receiving dostarlimab. Instead, in 2022, 23/28 (82.1%) centers administrated dostarlimab; of these, most had one to five patients in treatment (19/28, 67.9%) (**Figure 3**).

**Figure 4:**
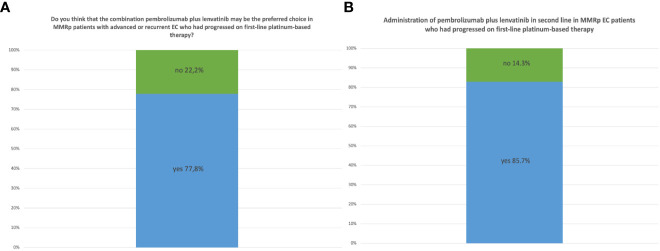
**(A)** Opinion of MITO centers in 2021 about the role of the combination of pembrolizumab plus lenvatinib as second-line therapy for pMMR patients. **(B)** Administration of pembrolizumab plus lenvatinib as second-line therapy in pMMR EC patients in MITO centers in 2022.

In 2021, we asked the participants what they believed to be the preferred second-line treatment for pMMR patients with advanced or recurrent EC who had progressed from first-line platinum-based therapy. Most (35/45, 77.8%) affirmed that the combination of pembrolizumab plus lenvatinib was going to become the preferred choice (**Figure 4A**).

In 2022, after the reporting of KEYNOTE-775 results (12), 24/28 (85.7%) centers already administrated the pembrolizumab plus lenvatinib combination in this setting of patients (**Figure 4B**).

We asked if the availability of a therapeutic option for dMMR/MSI-H patients has modified the diagnostic approach. Most (18/28, 64.3%) stated that universal molecular screening for all patients with a new diagnosis of EC was already done at their centers. The same question had been asked in the previous survey, where universal molecular screening in all patients with a new diagnosis of EC was performed in a lower percentage (43.2%). Once a deficiency in MMR machinery was detected on the tumor specimen, genetic counseling was planned before the blood sampling for the germline testing in 23/28 (82.1%) centers compared to 48.9% of the previous survey.

## 4 Discussion

With this survey, we aimed to evaluate how the use of molecular classification and the introduction of checkpoint inhibitors have changed the clinical approach in patients with advanced EC in Italy.

Molecular-based classification in EC should be performed using immunohistochemical markers (p53, MLH1, MSH2, MSH6, and PMS2) and one molecular test, the mutation analysis of the exonuclease domain of POLE, to identify prognostic groups (6, 17, 18).

It plays an important role in defining the indications for adjuvant treatment especially in the context of high-grade and/or high-risk EC (19). The application of molecular classification has revealed the existence of two groups: those with an excellent prognosis that are POLE mut tumors, and those with a poor prognosis that have p53-abnormal tumors. Endometrial carcinomas with dMMR or non-specific molecular profiles (NSMP) have an intermediate prognosis (20, 21). Moreover, germline mutations of one of the MMR genes (MLH1, PMS2, MSH2, and MSH6) are found in approximately 3% of all EC and approximately 10% of dMMR/MSI EC (22).

Testing for MMR/MSI status in patients with EC is relevant for several reasons. Firstly, dMMR/MSI is considered a marker for endometrioid-type EC, which is important for diagnostic purposes (23). Additionally, MMR testing is essential for pre-screening to identify patients who are at a higher risk of having Lynch syndrome, as well as for prognostic factors identified by TCGA (6). Furthermore, identifying patients who could benefit from treatment with immune checkpoint inhibitor therapy is also important (23).

The International Society of Gynecological Pathology (ISGyP) recommends testing for MMR status/MSI in all samples of endometrial carcinoma, regardless of the patient’s age (24, 25).

The most cost-effective method for identifying patients at high risk of Lynch syndrome is MMR-IHC on well-preserved tumor tissue. MMR-IHC is a reliable way to determine MMR status and to obtain information about the altered gene or protein (24). Therefore, the ISGyP guidelines recommend MMR-IHC as the preferred test (24). This test involves assessing the expression of four MMR proteins (MLH1, PMS2, MSH6, and MSH2) (26–28).

It is promising how the possibility of performing molecular classification in EC patients in Italian centers had significantly increased in just 1 year (75% versus 55.6%) as well as the possibility of carrying out molecular screening for all patients at time of diagnosis (64.3% versus 43.2%).

However, not all centers, at the time of survey, carried out all types of molecular test.

The mutation analysis of the exonuclease domain of POLE was performed only in 6/28 (21.4%) of centers, and MLH1 promoter methylation assessment was carried out by only 4/28 (14.3%) of participants.

The current utilization rates for these drugs in MITO centers are satisfactory, being 82.1% for dostarlimab and 85.7% for pembrolizumab plus lenvatinib.

Over the past year, the rise in the performance of molecular screening and the approval of dostarlimab’s reimbursement in Italy, compared to the previous compassionate use, which required more complicated procedures, have been accompanied by an increase in the prescription of checkpoint inhibitors.

The most important limitation of our survey is the low number of MITO members who answered the questionnaires, which have the same possible selection biases of the previous survey, with a high response rate from the centers of northern Italy. Another limitation of this multicenter study is that it includes several high-volume centers with different expertise, which could impact the results. The non-response rate to the questionnaire should be considered, as some centers likely have a lower volume of patients, potentially influencing their participation. In addition, this study did not compare responses between oncologists and gynecologists regarding checkpoint inhibitor use and molecular classification. Evaluating potential differences by specialty could be an area for future research as well as re-surveying centers in the future may be useful to continue monitoring developments in this rapidly evolving field.

In conclusion, considering the survey results, alongside with the comparison to the previous survey, the management for metastatic EC has changed both from a diagnostic and therapeutic point of view. These findings highlight the potential impact of recent diagnostic and therapeutic developments on clinical practice.

## Data availability statement

The raw data supporting the conclusions of this article will be made available by the authors, without undue reservation.

## Author contributions

GV, GG, and FA: Conceptualization; all authors: resources; FA, GG, and DC: Data curation; GG, FA, and GV: Formal analysis, software, and methodology; GC: Funding acquisition; GG, FA, and GV: Investigation and project administration; GV and GG: Supervision, validation, and visualization; GG, FA, and GV: Drafting of the manuscript; GG, GV, and FA: Review and editing; all authors. All authors contributed to the article and approved the submitted version.
