# Corrigendum: Management of metastatic endometrial cancer: physicians’ choices beyond the first line after approval of checkpoint inhibitors

**DOI:** 10.3389/fonc.2023.1357793

**Published:** 2024-01-22

**Authors:** Francesca Arezzo, Gaia Giannone, Daniele Castaldo, Giulia Scotto, Valentina Tuninetti, Margherita Turinetto, Michele Bartoletti, Serafina Mammoliti, Grazia Artioli, Giorgia Mangili, Vanda Salutari, Domenica Lorusso, Gennaro Cormio, Vera Loizzi, Claudio Zamagni, Antonella Savarese, Massimo Di Maio, Graziana Ronzino, Carmela Pisano, Sandro Pignata, Giorgio Valabrega

**Affiliations:** ^1^ Gynecologic Oncology Unit, IRCCS Istituto Tumori “Giovanni Paolo II”, Bari, Italy; ^2^ Department of Precision and Regenerative Medicine – Ionian Area, University of Bari “Aldo Moro”, Bari, Italy; ^3^ Department of Surgery and Cancer, Imperial College London, London, United Kingdom; ^4^ Segreteria Multicenter Italian Trials in Ovarian Cancer and Gynecologic Malignancies (MITO) Group, Naples, Italy; ^5^ Department of Oncology, University of Turin, Turin, Italy; ^6^ Department of Oncology, Mauriziano Hospital, University of Turin, Turin, Italy; ^7^ Unit of Medical Oncology and Cancer Prevention, Department of Medical Oncology, Centro di Riferimento Oncologico di Aviano (CRO), IRCCS, Aviano, Italy; ^8^ Ospedale Policlinico San Martino - Department of Medical Oncology, IRCCS, Genoa, Italy; ^9^ Oncologia Medica, Unità locale socio sanitaria n2 (ULSS2) Marca Trevigiana, Treviso, Italy; ^10^ Obstet-Gynecol Department, San Raffaele Scientific Institute, IRCCS, Milan, Italy; ^11^ Department of Women and Child Health, Division of Gynecologic Oncology, Fondazione Policlinico Universitario A. Gemelli, IRCCS, Rome, Italy; ^12^ Department of Life Science and Public Health, Catholic University of Sacred Heart Largo Agostino Gemelli, and Fondazione Policlinico Universitario A. Gemelli, IRCCS, Rome, Italy; ^13^ Interdisciplinar Department of Medicine, University of Bari “Aldo Moro”, Bari, Italy; ^14^ Azienda Ospedaliero-universitaria di Bologna, IRCCS, Bologna, Italy; ^15^ Department of Oncology, IRCCS Regina Elena National Cancer Institute, Rome, Italy; ^16^ Department of Oncology, Ospedale “Vito Fazzi”, Lecce, Italy; ^17^ Department of Urology and Gynecology, Istituto Nazionale Tumori IRCCS Fondazione G. Pascale Napoli, Naples, Italy

**Keywords:** endometrial cancer, molecular classification, immune checkpoint inhibitors, dostarlimab, pembrolizumab plus lenvatinib

In the published article, there was an error in affiliation 15. Instead of “Department of Oncology, Regina Elena National Cancer Institute, Rome, Italy”, it should be “Department of Oncology, IRCCS Regina Elena National Cancer Institute-Rome, Italy.”

The authors apologize for this error and state that this does not change the scientific conclusions of the article in any way. The original article has been updated.

