# Supplementary material for: Management of metastatic endometrial cancer: physicians’ choices beyond the first line after approval of checkpoint inhibitors
Source: Front Oncol. 2023 Sep 14;13:1247291. doi: 10.3389/fonc.2023.1247291 (PMC10538538; doi:10.3389/fonc.2023.1247291)
Supplement: Supplementary file 1 [file Table_1.docx]

Supplementary Material

# Management of Metastatic Endometrial Cancer: Physicians' Choices Beyond the First Line after approval of checkpoint inhibitors.

Francesca Arezzo ^1,2^, Gaia Giannone ^3,*^, Daniele Castaldo ^4^, Giulia Scotto^5^, Valentina Tuninetti^6^, Margherita Turinetto ^5^,Michele Bartoletti ^7^, Serafina Mammoliti^8^, Grazia Artioli ^9^, Giorgia Mangili^10^, Vanda Salutari ^11^, Domenica Lorusso^12^, Gennaro Cormio ^1,13^, Vera Loizzi ^1,13^, Claudio Zamagni ^14^, Antonella Savarese ^15^, Massimo Di Maio ^6^, Graziana Ronzino ^16^, Carmela Pisano ^17^, Sandro Pignata ^17^ and Giorgio Valabrega ^6^

*** Correspondence:** Gaia Giannone: [g.giannone@imperial.ac.uk](mailto:g.giannone@imperial.ac.uk)

Table S1: MITO questionnaire (translated into English).

| **Questions:** |
| --- |
| How old are you? |
| For how many years you have been working in the setting of gynecology oncology? |
| In what type of Hospital do you work? |
| In which area of Italy do you work? |
| Which is your speciality? |
| Do you manage above all gynaecologic malignancies/ only gynaecologic malignancies/other cancer types and marginally gynaecologic malignancies? |
| How many new Endometrial cancers (EC) are diagnosed in your centre in one month? |
| How many patients with recurrent/locally advanced not amenable for surgical treatment/metastatic EC are managed in your centre in one month? |
| How many patients with pretreated metastatic EC are managed in your centre in one month? |
| Do you have any clinical trial in II line for pretreated advanced EC patients? |
| Do you perform Estrogen/Progesterone Receptor immunohistochemistry (IHC) for EC patients’ samples? |
| Do you use the molecular classification for EC patients in everyday practice? |
| Which assays are performed at diagnosis in EC patients in your centre? (Among IHC for MSH6 and PMS2; IHC for MLH1, MSH2, MSH6 and PMS2; Polymerase chain reaction (PCR) for MSI; IHC for p53, POLE hotspot sequencing; MLH1 promoter methylation or others) |
| Which was your preferred treatment in II line for advanced Mismatch repair deficient (MMRd) patients? |
| How many MMRd patients are receiving dostarlimab in II line in your centre now? |
| Which was your preferred treatment in II line for Mismatch repair proficient (MMRp) advanced EC patients? |
| How many MMRp patients are receiving pembrolizumab + lenvetinib in II line in your centre now? |
| Which is your preferred treatment in III line for advanced MMRp patients? |
| Which is your preferred treatment in III line for advanced MMRd patients? |
| Regarding your experience with dostarlimab, how many patients, due to the occurrence of toxicity, required treatment discontinuation/dose reduction/hospitalization? |
| Regarding your experience with pembrolizumab, how many patients, due to the occurrence of toxicity, required treatment discontinuation/dose reduction/hospitalization? |
| Regarding your experience with the association pembrolizumab plus lenvatinib, how many patients, due to the occurrence of toxicity, required treatment discontinuation/dose reduction/hospitalization? |
| What do you believe could become the treatment of choice in I line for MMRp patients with advanced EC? |
| Has the availability of a therapeutic option for dMMR patients changed the diagnostic algorithm in your center? |
| Which are the subsequent steps after a diagnosis of MMRd EC? How do you organize genetic referrals in your centre? |

Table S2. MITO members percentage of response.

|  |  |
| --- | --- |
| Invitation to fill in the survey | 166 MITO centers, 708 MITO members |
| Invitation opened by | 372 clinicians (53%) |
| Link clicked on by | 69 clinicians (10%) |
| Survey completed by | 35 clinicians (4.9%) |
